# PPAR*γ* Plays an Important Role in Acute Hepatic Ischemia-Reperfusion Injury via AMPK/mTOR Pathway

**DOI:** 10.1155/2021/6626295

**Published:** 2021-07-03

**Authors:** Liwei Wu, Qiang Yu, Ping Cheng, Chuanyong Guo

**Affiliations:** ^1^Department of Gastroenterology, Shanghai Tenth People's Hospital, Tongji University School of Medicine, Shanghai 200072, China; ^2^Department of Gerontology, Shanghai Minhang District Central Hospital, Shanghai 201100, China

## Abstract

**Background:**

Hepatic ischemia-reperfusion (IR) injury is one of the severe complications associated with liver surgery and leads to liver dysfunction. PPAR*γ* is always linked with various physiologic pathways, and it can alleviate liver damage in IR injury.

**Aim:**

In this study, we explored the potential mechanism of PPAR*γ* in the pathogenesis of hepatic IR injury by mice model.

**Methods:**

After treated with si-PPAR*γ* or rosiglitazone, mice were subjected to hepatic ischemia-reperfusion. Liver tissue and blood samples were collected to evaluate liver injury and detected relative mRNA and protein expressions.

**Results:**

The expression of PPAR*γ* was increased after reperfusion. And the alleviation of PPAR*γ* aggravated the liver damage in IR; at the same time, upregulation of the expression of PPAR*γ* released the liver damage. And these effects of PPAR*γ* in IR were related to the AMPK/mTOR/autophagy signaling pathway.

**Conclusion:**

PPAR*γ* plays an important role in hepatic IR injury at least partly via the AMPK/mTOR/autophagy pathway.

## 1. Introduction

Ischemia-reperfusion (IR) is a phenomenon occurring after the restoration of arterial blood flow to a specific organ or tissue [1]. The pathophysiology of IR injury is mainly the induction of oxidative stress and inflammatory cascade reaction. Thus, the reperfusion of blood flow may result in oxidative damage and inflammation rather than recovery. Hepatic IR injury is one of the severe complications associated with liver surgery and leads to liver failure or primary nonfunction, thus, increasing morbidity and mortality after liver surgery [2–4]. Since hepatectomy or liver transplantation is the most effective method for the treatment of end-stage liver diseases, it is essential to detect the possible preoperative and perioperative interventions for minimizing IR-induced hepatocellular damage, especially in patients with cirrhosis.

As a member of the family of nuclear receptors, peroxisome proliferator-activated receptor-*γ* (PPAR*γ*) acts as heterodimers with DNA response elements and can regulate various metabolism responses [5, 6]. PPAR*γ* has an important role in regulating the inflammatory response and oxidative stress in hepatic IR injury [7–9]. The protecting effects of PPAR*γ* agonists, such as telmisartan [10], irbesartan [11], darglitazone [12], rosiglitazone [13], and pioglitazone [14], in IR injury have been reported [15]. And these evidences suggested that PPAR*γ* agonists can modulate inflammatory responses, oxidative stress, and metabolism in IR. The adenosine monophosphate-activated protein kinase/mammalian target of rapamycin (AMPK/mTOR) signaling pathway has been confirmed that it is a critical regulator of cellular processes, including cell growth, viability, differentiation, survival, and metabolism [16–18]; mTOR has been also identified as a key modulator of autophagy [19]; and its dysregulation has been implicated in a variety of pathological disorders, including playing critical roles in regulating liver IR injury. And PPAR*γ* can modulate the mTOR pathway. In this study, we downregulated and upregulated the expression of PPAR*γ* and explored the function of PPAR*γ* in hepatic IR injury, and we treated mice with mTOR inhibitor, rapamycin (Rapa), to make sure PPAR*γ* showed its effects in hepatic IR injury via AMPK/mTOR pathway.

## 2. Methods

### 2.1. Animal Preparation

This project was approved by the Animal Care and Use Committee of Shanghai Tongji University and Shanghai Tenth People's Hospital (SHDSYY-2021-4990), China. And all animal experiments complied with the guidelines of the China National Institutes of Health. Six-week-old male Balb/c mice (Shanghai SLAC Laboratory Animal, Shanghai, China) were used in our experiments. All mice weighed 23-28 g and housed in a standard environment. All efforts have been done to minimize the suffering of mice in this research.

The animals underwent either sham surgery or ischemia-reperfusion (IR) operation. Partial warm hepatic ischemia was induced as described previously [20]. After anesthetized with 1.25% sodium pentobarbital (Nembutal, St. Louis, MO, USA), the blood supply to the left lateral and median lobes of the liver was interrupted, causing 70% ischemia. After 45 minutes of hepatic ischemia, we restored blood supply and initiated reperfusion. We performed the same operation protocol in sham control mice without vascular occlusion. Mice were sacrificed after 2, 6, 12, and 24 hours of ischemia-reperfusion, and blood and liver were collected for further analysis.

Rapamycin (S1039, Selleck) was dissolved in dimethyl sulfoxide (DMSO) at 25 mg/ml before administration. In the rapamycin-treated groups, animals have received rapamycin at a dose of 1.5 mg/kg per day before injury through intraperitoneal injection.

### 2.2. *In Vivo* Transfection with PPAR*γ* Short Interfering RNA (siRNA)

Firstly, siRNA PPAR*γ* (guide: 5′UCAGCUCCGUGGAUCUCUCCGUAAU′, passenger: 5′AUUACGGAGAGAUCCACGGAGCUGA′) or siRNA control (GenePharma, Suzhou, China) was bought from Genema. Then, according to the producer instruction, siRNA PPAR*γ* or siRNA control was dissolved in RNase-free water to the concentration of 1 *μ*g/*μ*L. Then, 5 *μ*L PPAR*γ* siRNA or 5 *μ*L control siRNA and 5 *μ*L in vivo transfection reagent (18668-11, Engreen, Co., Beijing, China) were, respectively, diluted with 5 *μ*L 10% glucose. Finally, the mixtures were injected into the tail vein of mice.

### 2.3. Animal Experiment Design

According to our experiment plan, mice were distributed into the following groups:

1. Natural group (*n* = 3): mice without any treatment
2. Sham group (*n* = 5): mice underwent sham surgery
3. Vehicle group (*n* = 5): mice were treated with methylcellulose orally once a day for 5 days without operation
4. Drug group (*n* = 5): mice were treated with 10 mg/kg rosiglitazone orally once a day for 5 days without operation
5. Si-control group (*n* = 5): mice were injected from the tail vein with control siRNA once a day for 2 weeks without operation
6. Si-PPAR*γ* group (*n* = 5): mice were injected from the tail vein with PPAR*γ* siRNA once a day for 2 weeks without operation
7. IR groups (*n* = 20): mice underwent IR operation and sacrificed at 2, 6, 12, and 24 hours after reperfusion
8. IR + Rosi groups (*n* = 20): after treated with 10 mg/kg rosiglitazone orally once a day for 5 days, mice underwent IR operation and sacrificed at 2, 6, 12, and 24 hours after reperfusion
9. IR + si-PPAR*γ* groups (*n* = 20): after injected with PPAR*γ* siRNA once a day for 2 weeks, mice underwent IR operation and sacrificed at 2, 6, 12, and 24 hours after reperfusion
10. IR + si-PPAR*γ*+Rosi groups (*n* = 20): after treated with both PPAR*γ* siRNA and 10 mg/kg rosiglitazone, mice underwent IR operation and sacrificed at 2, 6, 12, and 24 hours after reperfusion
11. IR + Rapa (*n* = 5): after intraperitoneally injected with the mTOR inhibitor rapamycin for 5 days, mice underwent IR operation and sacrificed at 12 hours after reperfusion
12. IR + si-PPAR*γ*+Rapa (*n* = 5): after treated with both PPAR*γ* siRNA and rapamycin, mice underwent IR operation and sacrificed at 12 hours after reperfusion

### 2.4. Serum Enzyme Analysis

Serum was separated by centrifugation at 1,500 g for 10 min and stored at -80°C. Serum levels of aspartate aminotransferase (AST) and alanine aminotransferase (ALT) were measured by kits bought from Jiancheng Co. (Nanjing, China).

### 2.5. Histology, Immunohistochemical(IHC) Staining, and Terminal Deoxynucleotidyl Transferase dUTP Nick End Labeling (TUNEL) Assay

Liver tissue samples were fixed and embedded in the following standard steps. Liver sections were cut and stained with hematoxylin and eosin. For IHC, the slices were dewaxed and rehydrated; and after an antigen retrieval process and blocking, slices were incubated with primary antibodies described in western blot analysis part overnight. For the TUNEL, the slices were treated according to the instruction and then incubated in the TUNEL reaction mixture (Roche, Mannheim, Germany) at room temperature for 1 h.

### 2.6. Western Blot Analysis

Western blotting was performed as standard protocol. Protein was extracted from frozen liver samples. A total of 80 ng protein was loaded onto 6%, 10%, and 12.5% SDS-polyacrylamide gels, and the separated proteins were transferred to PVDF membranes. The membranes were incubated overnight at 4°C with primary antibodies followed by incubation with a secondary antibody (1 : 2,000). Finally, the blots were scanned using an Odyssey two-colour infrared laser imaging system (Li-Cor, Lincoln, NE, USA). Western blots were performed using the following antibodies; PPAR*γ* (Cell Signaling Technology), mTOR (Cell Signaling Technology), p-mTOR (Cell Signaling Technology, Ser2448), AMPKɑ (Cell Signaling Technology), p-AMPKɑ (Cell Signaling Technology, Thr172), TNF-ɑ (Cell Signaling Technology), IL-1*β* (Cell Signaling Technology), Bax (Proteintech), cleaved caspase-9 (Proteintech), Beclin1 (Proteintech), LC3 (Proteintech), and *β*-actin (Abcam).

### 2.7. RNA Extraction and Quantitative Real-Time- (qRT-) PCR Analysis

The total RNA was isolated from tissues according to the standard protocol. The first strand of cDNA was synthesized using a reverse transcription kit (TaKaRa Biotechnology) and was used to analyse the indicator expression. Real-time PCR experiments were performed according to the protocol of the real-time PCR kit (Takara, Otsu, Shiga, Japan). The ratio of each gene compared to *β*-actin was calculated by standardizing the ratio of each control to the unit value. The primer sequences for qRT-PCR were shown in Table 1.

### 2.8. Statistics

All experiments were conducted three times and were analyzed using Graph Pad Prism 5.0 software. Data are expressed as means ± SD. The differences between before and after IR of mice, with or without si-PPAR injection, and with or without TZDs administration were evaluated using two-way ANOVA with the Student's *t*-test to compare between the two groups. *p* value of less than 0.05 was considered statistically significant.

## 3. Results

### 3.1. The Expression of PPAR*γ* in IR Injury

**Figure 1:**
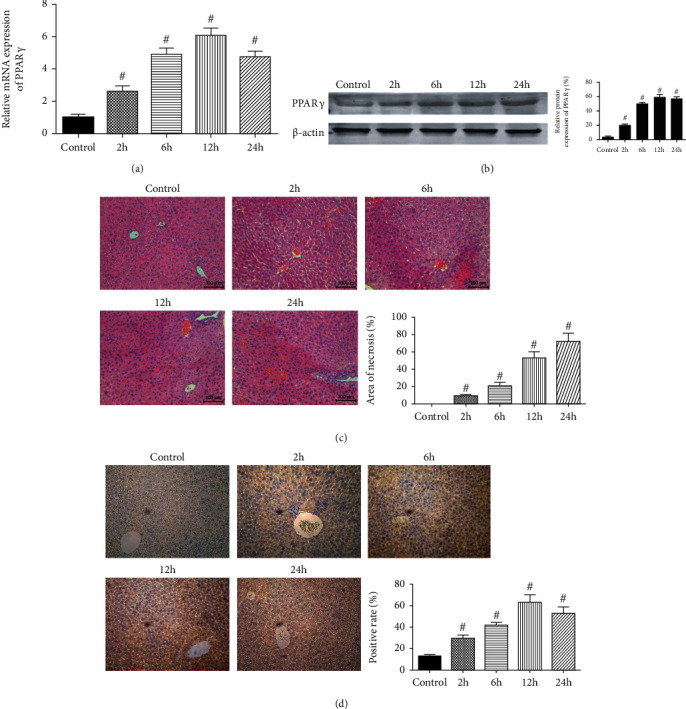
The expression of PPAR*γ* in IR injury. Notes: (a) relative mRNA expression of PPAR*γ* (*n* = 5, # means *p* < 0.05 for IR versus control after 2, 6, 12, and 24 h); (b) protein expression of PPAR*γ* (*n* = 3, # means *p* < 0.05 for IR versus control after 2, 6, 12, and 24 h); (c) representative H&E stained sections of the liver (original magnification, ×200). The ratio of necrosis area to total area was analyzed with Image-Pro Plus 6.0 (*n* = 5, # means *p* < 0.05 for IR versus control after 2, 6, 12, and 24 h); (d) Immunohistochemistry staining (×200) showing the expression of PPAR*γ*. The ratio of brown area to total area was analyzed with Image-Pro Plus 6.0 (*n* = 5, # means *p* < 0.05 for IR versus control after 2, 6, 12, and 24 h).

To confirm the activation of PPAR*γ* during the hepatic ischemia-reperfusion injury, we detected the expression of PPAR*γ* by western blot and qRT-PCR. In Figures 1(a) and 1(b), both the protein and mRNA expression of PPAR*γ* were increased after reperfusion, especially after 6 hours. Following, we did a histopathological analysis for IR injury (Figure 1(c)). Obvious necrosis could be seen after 6 hours, and the rate of necrosis was over 50% after 12 hours after reperfusion.  Figure 1(d) exhibited the immunohistochemical staining of PPAR*γ* in collected liver tissues, and the number of positive cells changed almost in parallel with the above results. We hypothesized that this change was due to the protective mechanism of PPAR*γ* on damaged hepatocytes. And we compared natural group, sham group, vehicle group, drug group, and siRNA-control groups to exclude their influence on the following results (Supplementary Figure 1).

### 3.2. Alleviation the Expression of PPAR*γ* Aggravated the Liver Damage in IR

**Figure 2:**
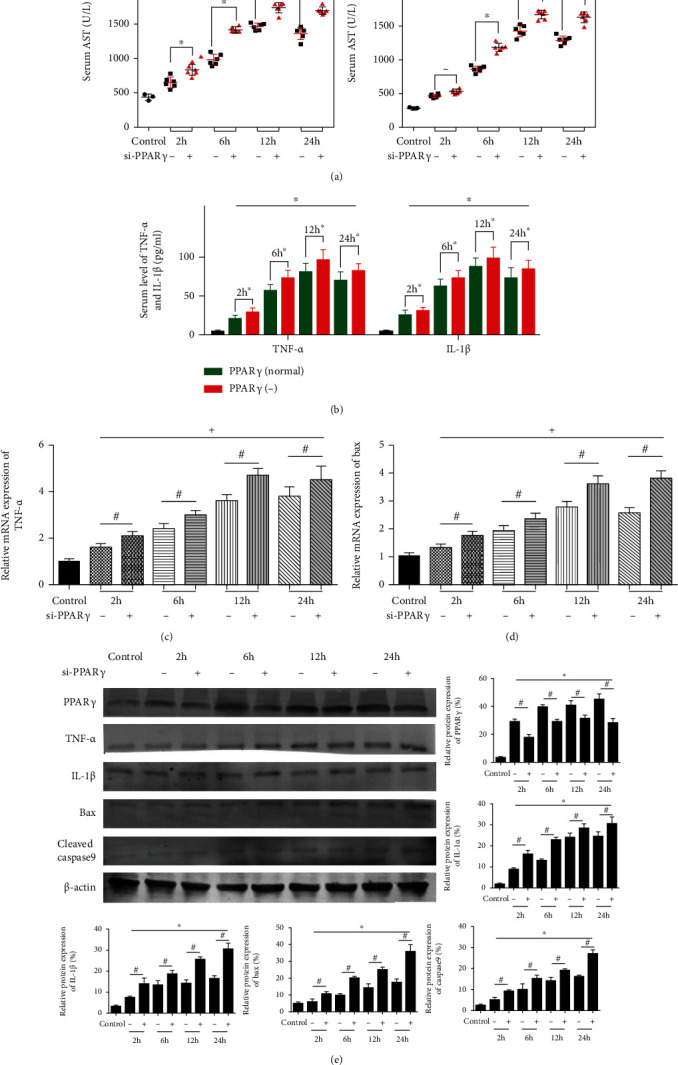
Effect of PPAR*γ* alleviation on liver function and pathology of mice in IR. Notes: (a) the levels of serum ALT and AST (*n* = 5, ∗ means *p* < 0.05 for si-PPAR*γ* versus IR after 2, 6, 12, and 24 h, + means *p* < 0.05 for IR verse control); (b) the levels of serum TNF-*α* and IL-1*β* (*n* = 5, ∗ means *p* < 0.05 for si-PPAR*γ* versus IR after 2, 6, 12, and 24 h, + means *p* < 0.05 for IR verse control); (c) relative mRNA expression of TNF-*α* (*n* = 5, # means *p* < 0.05 for si-PPAR*γ* versus IR after 2, 6, 12, and 24 h, + means *p* < 0.05 for IR verse control); (d) relative mRNA expression of Bax (*n* = 5, # means *p* < 0.05 for si-PPAR*γ* versus IR after 2, 6, 12, and 24 h, + means *p* < 0.05 for IR verse control); (e) protein expression of PPAR*γ*, TNF-*α*, IL-1*β*, Bax, and cleaved caspase9 (*n* = 3, # means *p* < 0.05 for si-PPAR*γ* versus IR after 2, 6, 12, and 24 h, ∗ means *p* < 0.05 for IR verse control).

Si-PPAR*γ* was injected into mice via the tail vein to downregulate its expression, and the pathological alteration after reperfusion was compared with that in normal mice. Serum levels of ALT and AST were measured after reperfusion (Figure 2(a)), and we found that the downregulation of PPAR*γ* aggravated the damage of hepatocytes. And then we evaluated the damage in terms of inflammation and apoptosis.

We detected the level of inflammation through proinflammatory cytokine TNF-ɑ and IL-1*β*. The circulating levels of them were indeed upregulated by PPAR*γ* downregulation (Figure 2(b)). Consistent with the former, the expression of TNF-ɑ and IL-1*β* was higher in the si-PPAR*γ* group (Figures 2(c) and 2(e)). Apoptosis is a prominent feature of IR injury, and its participation was confirmed. Bax is a famous proapoptotic family member, and we detected its mRNA expression firstly. The graph in Figure 2(d) showed that with the increase of Bax expression during IR injury, PPAR*γ* downregulation made this increase more obvious. Then, we used western blot to measure the protein expression of Bax and caspase9, whose results (Figure 2(e)) exhibited that the injection of si-PPAR*γ* increased the occurrence of apoptosis during IR injury. Thus, the alleviation of PPAR*γ* aggravated the liver damage in IR.

### 3.3. Upregulation of the Expression of PPAR*γ* Released the Liver Damage in IR

**Figure 3:**
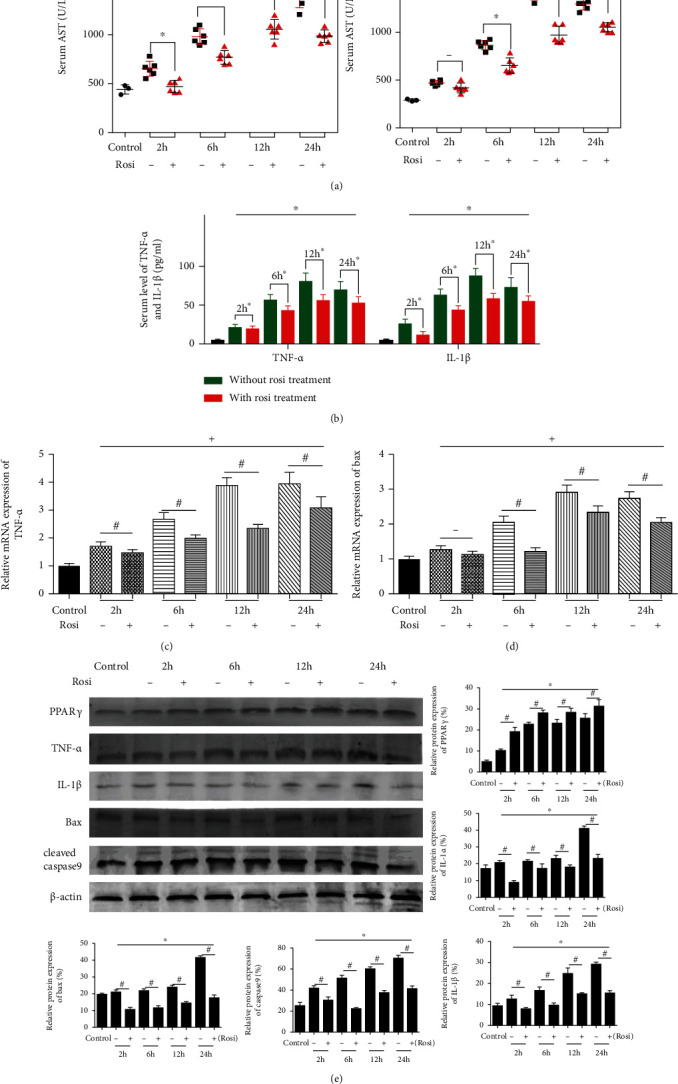
Effect of PPAR*γ* upregulation on liver function and pathology of mice in IR. Notes: (a) the levels of serum ALT and AST (*n* = 5, ∗ means *p* < 0.05 for Rosi versus IR after 2, 6, 12, and 24 h, + means *p* < 0.05 for IR verse control); (b) the levels of serum TNF-*α* and IL-1*β* (*n* = 5, ∗ means *p* < 0.05 for Rosi versus IR after 2, 6, 12, and 24 h, + means *p* < 0.05 for IR verse control); (c) relative mRNA expression of TNF-*α* (*n* = 5, # means *p* < 0.05 for Rosi versus IR after 2, 6, 12, and 24 h, + means *p* < 0.05 for IR verse control); (d) relative mRNA expression of Bax (*n* = 5, # means *p* < 0.05 for Rosi versus IR after 2, 6, 12, and 24 h, + means *p* < 0.05 for IR verse control); (e) protein expression of PPAR*γ*, TNF-*α*, IL-1*β*, Bax, and cleaved caspase9 (*n* = 3, # means *p* < 0.05 for si-PPAR*γ* versus IR after 2, 6, 12, and 24 h, ∗ means *p* < 0.05 for IR verse control).

Rosiglitazone is a typical PPAR*γ* agonist and is widely used in clinics. A group of mice were treated with 10 mg/kg rosiglitazone orally for 5 days before IR, and we also compared their pathological alteration after reperfusion with that in normal mice.  Figure 3(a) showed the serum levels of ALT and AST, and we found that the upregulation of PPAR*γ* reduced the damage of hepatocytes. And then we evaluated the damage in the same way we used it before.

We detected the level of TNF-ɑ and IL-1*β*. The circulating levels of them were downregulated by rosiglitazone (Figure 3(b)). Consistent with the former, the expression of TNF-ɑ and IL-1*β* was lower in the rosiglitazone treatment group (Figures 3(c) and 3(e)). The graph in Figures 3(d) and 3(e) showed that the increased expression of Bax and caspase9 during IR injury was relieved by the treatment of rosiglitazone, that is, the occurrence of apoptosis during IR injury was reduced. Thus, the upregulation of PPAR*γ* mitigated liver damage in IR.

### 3.4. The Influence of PPAR*γ* in IR May Be Linked with AMPK/mTOR/Autophagy Signaling Pathway

**Figure 4:**
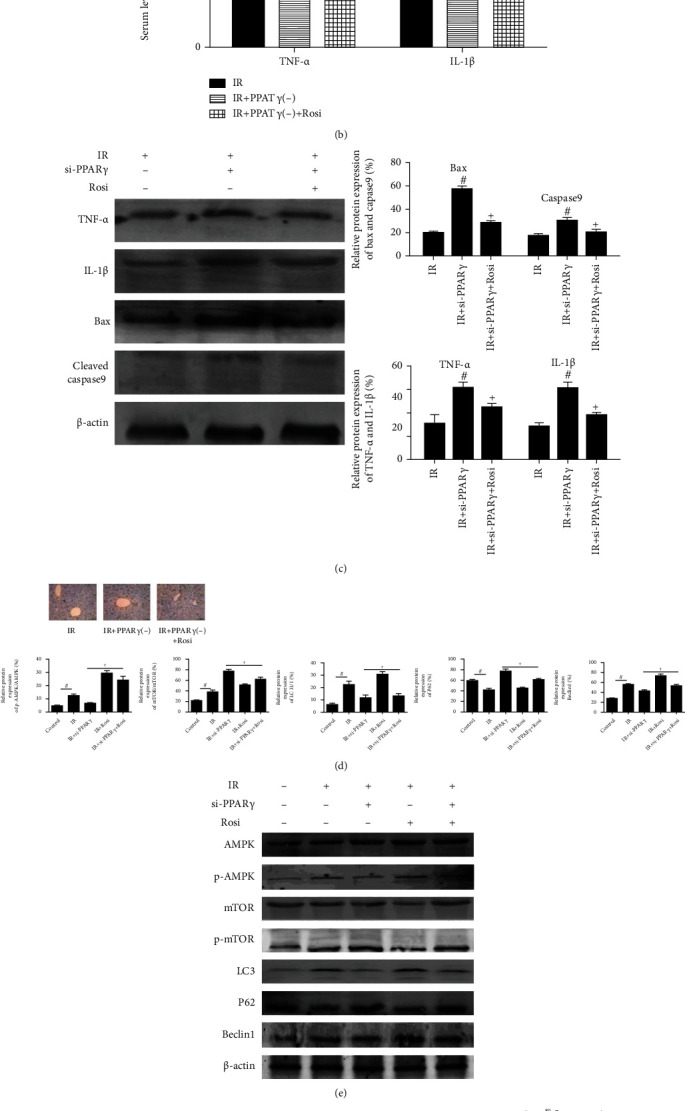
The influence of PPAR*γ* in IR may be linked with the AMPK/mTOR/autophagy signaling pathway. Notes: (a) the levels of serum ALT and AST for IR after 12 h (*n* = 5, # means *p* < 0.05 for si-PPAR*γ* versus IR, + means *p* < 0.05 for si-PPAR*γ*+Rosi versus si-PPAR*γ*); (b) the levels of serum TNF-*α* and IL-1*β* for IR after 12 h (*n* = 5, # means *p* < 0.05 for si-PPAR*γ* versus IR, + means *p* < 0.05 for si-PPAR*γ*+Rosi versus si-PPAR*γ*); (c) protein expression of TNF-*α*, IL-1*β*, Bax, and cleaved caspase9 for IR after 12 h (*n* = 3, # means *p* < 0.05 for si-PPAR*γ* versus IR, + means *p* < 0.05 for si-PPAR*γ*+Rosi versus si-PPAR*γ*); (d) TUNEL staining (×200) showed apoptotic cells in mice liver for IR after 12 h; (e) protein expression of AMPK, p-AMPK, mTOR, p-mTOR, LC3, and Beclin1 for IR after 12 h (*n* = 3, # means *p* < 0.05 for IR versus control, + means *p* < 0.05 for IR ± si-PPAR*γ*±Rosi versus IR); (f) immunohistochemistry staining (×200) showing the expression of p-AMPK, p-mTOR, and LC3 for IR after 12 h (*n* = 3, # means *p* < 0.05 for IR versus control, + means *p* < 0.05 for IR ± si-PPAR*γ*±Rosi versus IR).

To make sure the effect of PPAR*γ* in IR injury, those mice, which were injected with si-PPAR*γ*, were treated with Rosi. And we detected the index of apoptosis and inflammation in these mice after reperfusion for 12 hours, which were exhibited in Figures 4(a)–4(d). These results showed that liver damage during IR injury, including hepatocyte apoptosis and inflammation responses, was definitely related to the expression of PPAR*γ*. Besides, we also detected the changes of pyroptosis in our study, which were exhibited in Supplementary Figure 2.

As an important kinase in energy hemostasis, AMPK is an upstream target and negative regulator of mTOR, which is a major negative regulator of autophagy. Autophagy plays a vital role in various liver damage. Combined with previous reports, we speculated that the effects of PPAR*γ* during IR injury may be related to the AMPK/mTOR/autophagy signaling pathway. Thus, we measured the protein expression of p-AMPK, p-mTOR, and autophagy-related proteins, LC3, P62, and Beclin1, whose expressions were changed along with PPAR*γ* (Figures 4(e) and 4(f)). The treatment of Rosi obviously promoted AMPK phosphorylation and reduced the phosphorylated form of mTOR, which contributed to the suppression of autophagy in mouse livers. And si-PPAR*γ* leads to opposite effects. Furthermore, we detected the above changes in mice treated with both si-PPAR*γ* and Rosi, and the results were in agreement with those mentioned above. Thus, the effects of PPAR*γ* in IR injury were linked with the AMPK/mTOR/autophagy signaling pathway.

**Figure 5:**
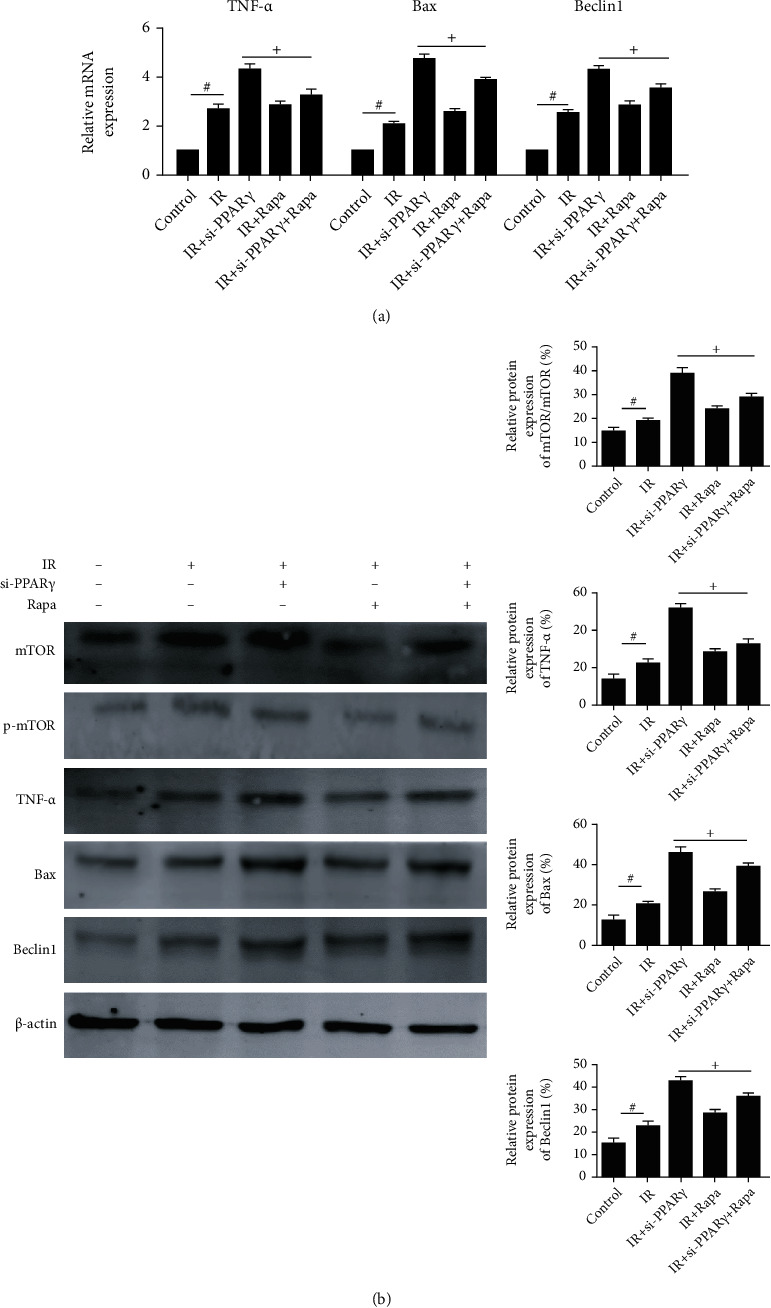
The involvement of AMPK/mTOR in the effects of PPAR*γ* in IR. Notes: (a) relative mRNA expression of TNF-*α*, Bax, and Beclin1 (*n* = 3, # means *p* < 0.05 for IR versus control, + means *p* < 0.05 for IR ± si-PPAR*γ*±Rapa versus IR); (b) protein expression of mTOR, p-mTOR, TNF-*α*, Bax, and Beclin1 (*n* = 5, # means *p* < 0.05 for si-PPAR*γ* versus IR, + means *p* < 0.05 for si-PPAR*γ*+Rosi versus si-PPAR*γ*).

To make sure our conclusion, we treated mice with rapamycin to inhibit the function of mTOR and detected changes in inflammation response and apoptosis, whose results were shown in Figure 5. We measured the expression of TNF-*α*, Bax, and Beclin1, and we found that effects caused by si-PPAR*γ* were blocked by rapamycin. Accordingly, we confirmed the relationship between PPAR*γ* and AMPK/mTOR.

## 4. Discussion

In the liver, IR injury can occur in several clinical situations, for example, liver trauma, resection, and transplantation. The pathogenesis of IR is closely related to oxidative stress, energy metabolism disorders, inflammatory response, and cell apoptosis and autophagy [21]. As is known to all, PPAR*γ* demonstrated significant functions in the tissue protection and repair [22–24]. And advances in PPAR ligands and agonists renew opportunities for drug development [25]. Here, in our present study, we found that the activation of PPAR*γ* could confer hepatoprotective effects against hepatic IR injury and also investigated the therapeutic potential of PPAR*γ* agonists for the protection of hepatic injury. The major findings of this study include (1) the expression of PPAR*γ* were increased after reperfusion, which hinted the protective role of PPAR*γ* in hepatic IR injury; (2) alleviation the expression of PPAR*γ* could aggravate the liver damage in IR; otherwise, the upregulation released liver damage; (3) the protective effects of PPAR*γ* may involve anti-inflammatory and antiapoptosis activity as demonstrated in vivo; (4) the underlying mechanism of PPAR*γ* in IR injury may be linked with AMPK/mTOR/autophagy signaling pathway.

PPAR*γ* is a ligand-activated transcription factor of the nuclear hormone receptor superfamily known to modulate target genes involved in the regulation of various inflammatory responses, cell growth and apoptosis, metabolism, fibrosis, and tissue repair [26, 27]. Several studies have indicated that the activation of PPAR*γ* is a therapeutic target for acute hepatic IR injury [7, 15, 28]. Additionally, agonists of PPAR*γ* exhibited anti-inflammation and antiapoptosis properties both in vitro and in vivo and could impart protection from IR in mice models [29, 30]. In the present experiment, our results demonstrated that the expression of PPAR*γ* was increased during reperfusion. Combined with the previous researches, we hypothesized that this change was due to the spontaneous protective mechanism of PPAR*γ* on damaged hepatocytes.

To clarify our hypothesis, we regulated the expression of PPAR*γ* by injection of si-PPAR*γ* or administration of rosiglitazone, a typical PPAR*γ* agonist. After the above treatment, serum liver enzymes ALT and AST showed the same changes as expected. And then we detected the liver damage in terms of inflammation and apoptosis. Excessive inflammatory response and hepatocyte apoptosis are recognized as key mechanism of liver IR injury. We detected the level of inflammation through proinflammatory cytokines TNF-ɑ and IL-1*β*. The circulating levels of them were obviously upregulated by si-PPAR*γ* and downregulated by rosiglitazone. Results of qRT-PCR and western blot exhibited that when compared with the IR group, the expression of TNF-ɑ and IL-1*β* was higher in the si-PPAR*γ* group and lower in the rosiglitazone group. Apoptosis is a prominent feature of IR injury, and its participation was confirmed. Bax and caspase9 are famous proapoptotic indices, and we detected their mRNA and protein expression. With the increase of Bax and caspase9 expression during IR injury, PPAR*γ* downregulation exacerbated this increase; however, the treatment of rosiglitazone relieved it. Thus, the alleviation of PPAR*γ* aggravated the liver damage in IR; and at the same time, the upregulation of PPAR*γ* mitigated the liver damage. Further, it has been reported that rosiglitazone is protective on a variety of injuries, including IR injury of many organs. Our results indicated that rosiglitazone may reduce, although do not ablate, hepatic damage after IR injury.

When further elucidating the potential mechanism of the effects of PPAR in IR, we focused on the pathway, AMPK/mTOR mediated autophagy. AMPK exists in all eukaryotic cells as a highly conserved protein kinase. It is a major regulator of energy homeostasis that balances energy supply and demand and ultimately modulates cellular and organ growth [31, 32]. AMPK can be activated by a variety of stresses including poisonous metabolites and pathological precursors such as starvation, ischemia, and hypoxia [33]. mTOR, a 289 kDa serine/threonine kinase, is a master negative regulator of autophagy, modulating cell growth, cell proliferation, cell cycle, and cell motility [16]. Autophagy plays a key role in the modulation of inflammation, cellular homeostasis and dysregulation, and cell death or survival. It has been proved associated with various liver disorders, including hepatitis, liver fibrosis, fatty liver, and acute IR injury [34–37]. It has been accepted that AMPK inhibits mTORC1 through phosphorylation, thus, inducing autophagy in response to cellular stress cues.

**Figure 6:**
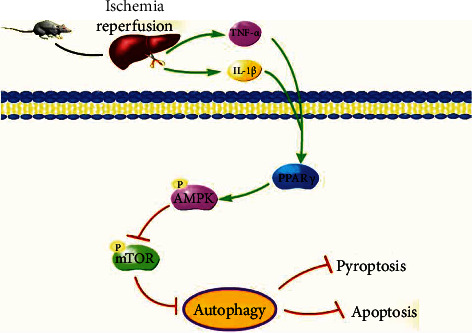
The underlying mechanism of PPAR*γ* in IR injury. Notes: activated PPAR*γ* in IR promoted AMPK phosphorylation and inhibited the phosphorylated form of mTOR, which contributed to the suppression of autophagy in mouse livers. And thus, PPAR*γ* exhibited its protective effects in the hepatic IR injury.

The relationship between PPAR*γ* and AMPK/mTOR pathway has been discussed before [17, 38]. Jimenez-Flores et al. [39] and Zhong et al. [40] reported that p-AMPK and PPAR-*γ* expression levels are significantly reduced in diabetic mouse livers and the increase of the expression of alleviated liver damage. Zhong et al. [41] found db/db mice showed significantly decreased PPAR-*γ* and p-AMPK expression levels and increased p-mTOR expression, and the expression of Atg7, Beclin-1, and LC3 was also decreased. Micheliolide reversed these effects and alleviated the inflammatory response and lipotoxicity in hepatocytes. Besides, the link of PPAR*γ* and AMPK/mTOR/autophagy pathway was explored in other disease models [42–48]. To make sure the mechanism, mice were treated with at least one of the si-PPAR*γ* and Rosi. The diminished expression of PPAR*γ* caused by si-PPAR*γ* leads to obvious inhibition of AMPK phosphorylation and thus promoted the phosphorylation of mTOR, inducing autophagy in mouse livers. The treatment of Rosi leads to opposite effects. Further, we treated mice with both si-PPAR*γ* and rosiglitazone and got the same result as expected. In addition, we used rapamycin to confirm the involvement of mTOR and found that inflammation response and apoptosis caused by rapamycin in the IR injury were changed opposite to si-PPAR*γ*. Therefore, we believed that the activation of PPAR*γ* can not only relieve the inflammatory response and hepatocyte apoptosis but also exert its hepatoprotective effect via the AMPK/mTOR/autophagy pathway (Figure 6).

## 5. Conclusion

In summary, we found that PPAR*γ* is continuously activated in hepatocytes during hepatic IR injury. Mice with significantly diminished expression of PPAR*γ* got more grievous liver injury after hepatic ischemia-reperfusion injury. Conversely, activation of PPAR*γ* caused by rosiglitazone resulted in attenuated liver injury. Through the use of si-PPAR*γ* and rosiglitazone, we confirmed that one possible mechanism by which PPAR*γ* activation results in protection against IR-related liver injury is through AMPK/mTOR-mediated autophagy. These results suggested that PPAR*γ* may be a vital regulator and potential therapeutic target in the liver ischemic injury. And our results provided confidence for the follow-up development of PPAR*γ*-related drugs for IR injury.

## Figures and Tables

**Table 1:** Sequences of primer pairs used for amplification of mRNA by real-time PCR.

	Forward	Reverse
*β*-Actin	GGCTGTATTCCCCTCCATCG	CCAGTTGGTAACAATGCCATGT
Bax	AGACAGGGGCCTTTTTGCTAC	AATTCGCCGGAGACACTCG
TNF-*α*	CAGGCGGTGCCTATGTCTC	CGATCACCCCGAAGTTCAGTAG
PPAR*γ*	GTCTTGGATGTCCTCGATGGG	TTATGGAGCCTAAGTTTGAGTTTGC

## Data Availability

The datasets generated during and/or analyzed during the current study are available from the corresponding author on reasonable request.

## Abbreviations

AST: Aspartate aminotransferase
ALT: Alanine aminotransferase
IHC: Histology, immunohistochemical staining
TUNEL: Terminal deoxynucleotidyl transferase dUTP nick end labeling assay
qRT-PCR: Quantitative real-time PCR analysis
IR: Ischemia-reperfusion
PPAR*γ*: Peroxisome proliferator-activated receptor-*γ*
AMPK: Adenosine monophosphate-activated protein kinase
mTOR: Mammalian target of rapamycin
Rosi: Rosiglitazone.

## Supplementary Materials

Supplementary Materials Supplementary Materials 1: comparison of control groups. We compared natural group, sham group, vehicle group, drug group, and siRNA-control groups to exclude their influence on the results. We detected serum ALT and AST levels of those groups and observed pathological changes of their liver tissues. Results were exhibited as follows. We found that there is no significant difference among these groups, which meant that they would not cause obvious influence in our study. 2: PPAR*γ* could affect pyroptosis in the IR injury. NOD-like receptor protein 3 (NLRP3) inflammasome plays a big role in the development of pyroptosis and can lead to the activation of caspase1. In the supplementary experiments, we measured the circulating level of IL-18 (a) and the mRNA and protein expression of caspase1 and NLRP3 among different groups of mice (b and c) and detected that relationship among IR, PPAR*γ*, and pyroptosis. We found that pyroptosis was increased in the IR, and the alleviation of PPAR*γ* aggravated pyroptosis, but the upregulation of PPAR*γ* released it.
