# Supplementary material for: PPARγ Plays an Important Role in Acute Hepatic Ischemia-Reperfusion Injury via AMPK/mTOR Pathway
Source: PPAR Res. 2021 Jul 3;2021:6626295. doi: 10.1155/2021/6626295 (PMC8275421; doi:10.1155/2021/6626295)

**PPARʏ plays an important role in Acute Hepatic Ischemia-Reperfusion Injury via AMPK/ mTOR pathway**

**Supplymentary Figures**

1. *Comparison of control groups*

We compared natural group,sham group,vehicle group,drug group, siRNA-control groups to exclude their influence on the results. We detected serum ALT and AST levels of those groups,and observed pathological changes of their liver tissues. Results were exhibited following. We found that there is no significant difference among these groups, which meant that they wouldn't cause obvious influence in our study.


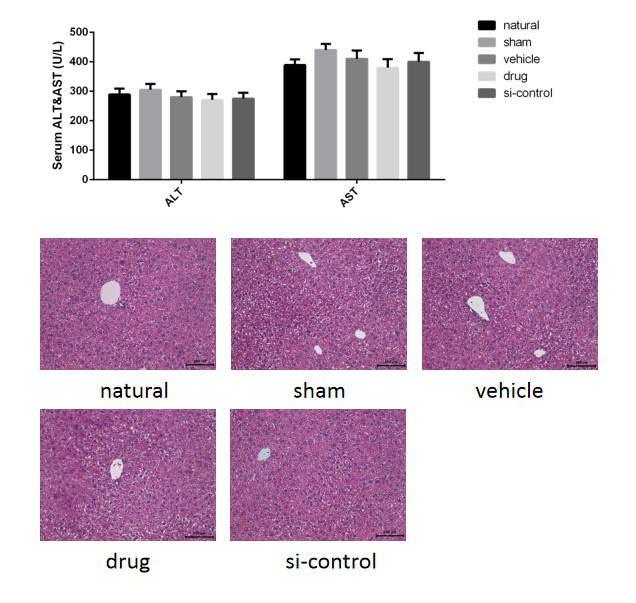


1. *PPARʏ could affect pyroptosis in the IR injury*

NOD-like receptor protein 3 (NLRP3) inflammasome plays a big role in the development of pyroptosis,and can lea to the activation of caspase1. In the supplementary experiments,we measured the circulating level of IL-18(A) and the mRNA and protein expression of caspase1 and NLRP3 among different groups of mice(B and C), and detected that relationship among IR, PPARʏ and pyroptosis. We found that pyroptosis was increased in the IR; and the alleviation of PPARʏ aggravated pyroptosis ,but the upregulation of PPARʏ released it.


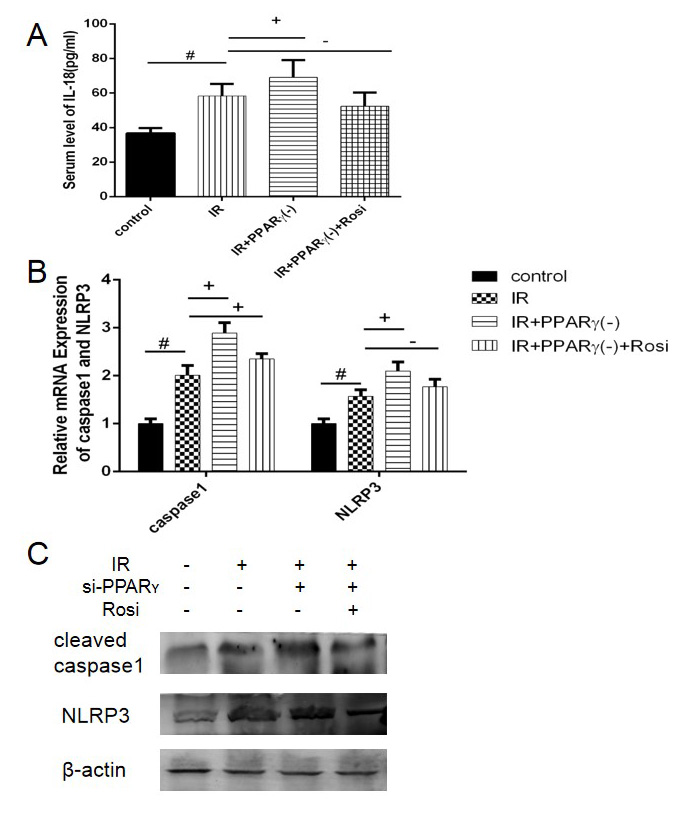

Supplement: Supplementary Materials — 1: comparison of control groups. We compared natural group, sham group, vehicle group, drug group, and siRNA-control groups to exclude their influence on the results. We detected serum ALT and AST levels of those groups and observed pathological changes of their liver tissues. Results were exhibited as follows. We found that there is no significant difference among these groups, which meant that they would not cause obvious influence in our study. 2: PPARγ could affect pyroptosis in the IR injury. NOD-like receptor protein 3 (NLRP3) inflammasome plays a big role in the development of pyroptosis and can lead to the activation of caspase1. In the supplementary experiments, we measured the circulating level of IL-18 (a) and the mRNA and protein expression of caspase1 and NLRP3 among different groups of mice (b and c) and detected that relationship among IR, PPARγ, and pyroptosis. We found that pyroptosis was increased in the IR, and the alleviation of PPARγ aggravated pyroptosis, but the upregulation of PPARγ released it. [file 6626295.f1.docx]
